# Sweetening the Food of Animals

**Published:** 1875-04

**Authors:** 


SWEETENING THE FOOD OF ANIMALS.—In fattening animals, time is often of importance to the feeder. Sometimes a month gained is equal to twenty per cent greater weight at a later period. Cooking food renders its constituents more soluble and digestible, therefore more rapidly entering on flesh and fat. As a condiment and appetizer for fattening animals, molasses has no equal. A small quantity of sweet, used upon hay, will cause a larger quantity to be eaten with a relish. We have often tried molasses upon poor animals with great satisfaction. A poor horse will show a change in condition in a few days. Molasses is not only an excellent condiment, but an excellent food; and being so soluble and assimilable that it produces an immediate effect upon the condition of the animal. Three pints may be fed to fattening animals per day; but to cows and breeding stock it must be fed sparingly, not more than a pint a day to a cow, as too much sweet will prevent their breeding. When necessary to use straw for fattening stock, the use of molasses diluted with eight or ten proportions of water, to wet the straw before steaming, will be found to render it very palatable, and cause it to be eaten, incorporated with other fattening food, as readily as hay. Some noted chemists have supposed all starchy food to be converted into sugar by the action of the stomach before it becomes assimilated as food. Perhaps this will account for the effects of sweet food upon animals.

---

The path to a true friend lies straight, though he be countless leagues away.

CONTENTMENT.—It is the greatest folly to set the heart upon uncertainties; and then, if disappointed, refuse to be comforted or reconciled. Do the very best you can, and then take things as they come. If a man strives with his best knowlege, energy, and untiring labor, to accomplish a certain object, working with skill and patience, he is a success, whether the scheme fails or succeeds, and he ought to reconcile himself to failure, if it was inevitable. If his labors have been of brain and hand, he is better fitted to succeed in other undertakings.

The secret of happiness is to make the best of everything; no matter what happens to annoy, let it all glide along as easily and with as few words of complaint and fault-finding as possible. Little inconveniences will intrude upon the most fortunate people, so the only way to be master of every situation is to make up your mind not to notice small annoyances. People may keep themselves in a constant broil over what amounts to nothing; and, without accomplishing the least good, may ruin the peace and quiet of a household. We cannot have everything just as we want it in this world, and the sooner a person understands that fact the sooner he may have a true basis for happiness.

---

PHOSPHORUS IN EYE DISEASE.—A case is reported to us of total blindness from cataract, attended with almost constant headaches, nearly cured in four months by dropping two or three drops of phosphorized oil into the eye daily, and using frictions of the same over the forehead. The opacity disappeared, and entire restoration appears probable.

